# Oestrogen receptor status and the response of human breast cancer cell lines to a combination of methotrexate and 17-beta oestradiol.

**DOI:** 10.1038/bjc.1985.48

**Published:** 1985-03

**Authors:** R. Clarke, H. W. Van den Berg, D. G. Kennedy, R. F. Murphy

## Abstract

We have investigated the modifying influence of 17-beta oestradiol (E2), on the cytotoxicity of methotrexate (MTX) towards two cell lines derived from human breast carcinoma. E2 (10(-7) M-10(-6) M) significantly reduced the antimetabolic effects of the drug towards an E2 non-responsive cell line, MDA-MB-436, whilst potentiating the action of MTX in an E2 responsive line, MCF-7. Similarly, E2 (10(-6) M) partially reversed the anti-proliferative effects of MTX in the MDA-MB-436 line and potentiated growth inhibition in the E2 responsive cells. This potentiation was not observed if E2 was replaced by the less biologically active alpha-isomer. In both cell lines pharmacological concentrations of the E2 reduced intracellular levels of MTX achieved during a 48 h treatment period. The latter finding is consistent with the ability of E2 to protect MDA-MB-436 cells from the action of MTX. Potentiation of the effects of MTX towards MCF-7 cells occurs despite reduced intra-cellular drug levels.


*Br. J. Cancer* (1985), **51**, 365–369

### Oestrogen receptor status and the response of human breast cancer cell lines to a combination of methotrexate and 17-*β* oestradiol

R. Clarke[1], H.W. Van den Berg[2], D.G. Kennedy[1] & R.F. Murphy[1]

*Departments of [1]Biochemistry and [2]Therapeutics & Pharmacology, The Queen's University of Belfast, 97 Lisburn Road, Belfast BT9 7BL, N. Ireland.*

**Summary** We have investigated the modifying influence of 17-*β* oestradiol (E2), on the cytotoxicity of methotrexate (MTX) towards two cell lines derived from human breast carcinoma. E2 ($10^{-7}$M – $10^{-6}$M) significantly reduced the antimetabolic effects of the drug towards an E2 non-responsive cell line, MDA-MB-436, whilst potentiating the action of MTX in an E2 responsive line, MCF-7. Similarly, E2 ($10^{-6}$M) partially reversed the anti-proliferative effects of MTX in the MDA-MB-436 line and potentiated growth inhibition in the E2 responsive cells. This potentiation was not observed if E2 was replaced by the less biologically active α-isomer. In both cell lines pharmacological concentrations of the E2 reduced intracellular levels of MTX achieved during a 48 h treatment period. The latter finding is consistent with the ability of E2 to protect MDA-MB-436 cells from the action of MTX. Potentiation of the effects of MTX towards MCF-7 cells occurs despite reduced intra-cellular drug levels.

Chemotherapy and oestrogen or anti-oestrogen therapy are important treatments for breast cancer. High response rates to endocrine manipulative therapy have been reported when treatment is based on oestrogen receptor status of the patient. Consideration of the heterogeneous nature of breast cancer with respect to steroid hormone receptor content suggests that combined hormone and cytotoxic drug therapy may be more beneficial than either treatment alone. The first trials designed to test the efficacy of combined therapy yielded conflicting results (Brunner *et al.*, 1977; Carter, 1981). Although the rationale for combined therapy is largely based on the hypothesis that endocrine therapy will selectively kill receptor positive cells whilst cytotoxic drug treatment will kill those cells uninfluenced by the hormonal environment, little is known of the modulating influence of hormones on the efficacy of cytotoxic drugs.

We have previously reported that pharmacological concentrations of 17-*β* oestradiol (E2), reduced the anti-metabolic and anti-proliferative effects of methotrexate (MTX) towards an E2 non-responsive human breast cancer cell line MDA-MB-436, an effect which correlated with an E2 induced reduction of intracellular MTX steady-state levels (Clarke *et al.*, 1983). These results contrast with an earlier report which demonstrated that the cytotoxicity of cytosine arabinoside towards the E2 responsive cell line MCF-7 was enhanced by physiological concentrations of the hormone (Weichselbaum *et al.*, 1978).

We have therefore extended our studies to include an investigation of the modulating influence of E2 on MTX toxicity towards the MCF-7 cell line.

### Materials and methods

### *Cell culture and treatment conditions*

MDA-MB-436 cells were obtained from Flow laboratories (Irvine, Scotland), and MCF-7 cells were the gift of Dr. C.D. Green, Liverpool University. MDA-MB-436 and MCF-7 cells were routinely maintained in Liebowitz L-15 and Eagles Modified Minimal Essential medium respectively. Both media were supplemented with 5% foetal calf serum, 100 IU ml$^{-1}$ penicillin and 100 $\mu$g ml$^{-1}$ streptomycin. Four days before treatment medium was replaced by medium containing 5% serum which had been stripped of endogenous steroids using dextran coated charcoal. Approximately $10^4$ cells were seeded into microwell culture dishes and exposed to E2 or solvent (ethanol, 10 $\mu$l) for 24 h prior to a further 24 h exposure to MTX in the absence or presence of the hormone. The antimetabolic effect of treatment was assessed by determination of incorporation of [$^3$H]-deoxyuridine into DNA as previously described (Van den Berg & Ball, 1972). Identically treated cells were assessed

Correspondence: H.W. Van den Berg.
Received 3 May 1984; and revised form 6 November 1984.

D

© The Macmillan Press Ltd., 1985

for their proliferative capacity by replacing drug containing medium with fresh medium with or without E2 and determining cell number after 6 days growth.

*Determination of intracellular MTX steady-state levels*

Intracellular levels of MTX were assessed during a 48 h exposure of cells to $10^{-7}$ M [$^3$H]-MTX (3,5,7-[3H]-MTX, Sp. act. 20 Ci mmol$^{-1}$, Amersham International). Cells$^{(5\times10^5)}$ were plated onto 5 cm petri dishes and exposed to labelled drug in the presence or absence of $10^{-6}$ M E2. At appropriate times cells were removed, washed ×4 with isotonic saline and the cell pellet dissolved in 5 mM Tris-HCl buffer, pH 7.4, containing 0.15 M NaCl, 10 mM EDTA, 0.5% (w/v) sodium dodecylsulphate and 0.02% sodium azide. Radioactivity was determined by scintillation counting and intracellular MTX expressed as fmol. MTX mg$^{-1}$ protein.

*Hormone receptor assays*

E2 receptor content was determined using dextran coated charcoal to separate free from receptor bound [$^3$H]-E2 (Shafie & Brookes, 1979). Progesterone receptor was assayed using the method of Pilchon & Milgrom (1977). (2,4,5,7-[$^3$H]) E2 (Sp. act. >90 Ci mmol$^{-1}$ and (1,2,6,7,16,17-[$^3$H]) progesterone (Sp. act. >100 Ci mmol$^{-1}$) were obtained from Amersham International.

### Results

Table I compares the E2 and progesterone receptor status of the MDA-MB-436 and MCF-7 cell lines. The former line synthesises low levels of E2 receptor which is detectable in both cytoplasm and nucleus. However E2 fails to induce the synthesis of progesterone receptor and the cells are non-responsive to E2 in terms of effects on DNA synthesis or cell proliferation (Clarke *et al.*, 1983). This line therefore appears to possess defects distal to the E2 receptor translocation step. Our results confirm that the MCF-7 cell line synthesises high levels of the E2 receptor which is functional by the criterion of mediating synthesis of the progesterone receptor. Furthermore exposure of MCF-7 cells to a wide range of E2 concentrations results in stimulation of incorporation of [$^3$H]-UdR into DNA (Figure 1).

Although [$^3$H]-UdR incorporation is stimulated in MCF-7 cells by E2, the hormone potentiated in a dose-dependent manner the ability of MTX to inhibit incorporation of this precursor into DNA

**Table I** Cytoplasmic oestrogen receptor (CER), nuclear oestrogen receptor (NER) and progesterone receptor (PGR) content of the MDA-MB-436 and MCF-7 cell lines.

| | *Cell line* | |
|---|---|---|
| *Receptor* | *MDA-MB-436* | *MCF-7* |
| CER | 12 | 251 |
| NER | 6 | 150 |
| PGR | 0 | 21 |

Values are fmol mg$^{-1}$ protein (Means of 3 determinations).

**Figure 1** The influence of a 24 h exposure to E2 on incorporation of [$^3$H]-deoxyuridine into DNA of MCF-7 cells. Results are the mean of 4 determinations.

(Figure 2). In marked contrast, the same figure shows that E2 exposure partially reversed MTX induced inhibition of [$^3$H]-UdR in the E2 non-responsive cell line. In both cell lines E2 modulation of the antimetabolic effects of MTX was significant at hormone concentrations of $10^{-7}$ and $10^{-6}$ M ($P<0.01$), Student's paired $t$ test). Figure 3 shows that modification of the antimetabolic effect of MTX ultimately led to similar modulation of the anti-proliferative action of the drug. The MDA-MB-436 cell line is intrinsically more sensitive to MTX than is the MCF-7 line. Concentrations of the drug were chosen for each line to result in approximately the same degree of growth inhibition. At the two concentrations used $10^{-6}$ M E2 significantly reversed the anti-proliferative effect of MTX towards the E2 non-responsive line whilst potentiating the drug's effect

**Figure 2** The effect of E2 on the inhibition of [$^3$H]-deoxyuridine incorporation resulting from a 24 h treatment with $10^{-8}$ M MTX, (●) (MDA-MB-436) and $5 \times 10^{-8}$ M MTX, (▲) (MCF-7). Results are the mean of 4 determinations.

on the E2 responsive line. In this experiment increased growth inhibition in the MCF-7 line was observed in the absence of any significant effect on growth by E2 alone.

We have previously shown that the reversal of the effects of MTX by E2 in the MDA-MB-436 line is accompanied by a reduction in the steady state level of the drug achieved intra-cellularly (Clarke *et al.*, 1983). Table II demonstrates that the intra-cellular levels of MTX are significantly reduced 24 and 48 h from start of drug exposure ($10^{-7}$ M) in both cell lines by $10^{-6}$ M E2. Preliminary studies have indicated that this effect of E2 may be related to the hormone's ability to induce a decrease in cell membrane fluidity as measured using a fluorescent probe (data not shown).

**Table II** The effect of $10^{-6}$ M E2 on the intra-cellular levels of MTX achieved during exposure of cells to $10^{-7}$ M [$^3$H]-MTX

| | | *Intra cellular MTX (fmol mg$^{-1}$ protein)* | |
|---|---|---|---|
| *Cell line* | | *24 h* | *48 h* |
| MCF-7 | *Control* | 3215±82 | 3593±130 |
| | $10^{-6}$ M E2 | 2647±69 | 2817±37 |
| | | ($P$<0.001) | ($P$<0.001) |
| MDA-MB-436 | *Control* | 1003±21 | 1345±54 |
| | $10^{-6}$ M E2 | 918±53 | 1005±49 |
| | | ($P$<0.01) | ($P$<0.001) |

**Figure 3** The effect of E2 ($10^{-6}$ M) on the antiproliferative action of MTX towards MDA-MB-436 and MCF-7 cells. Results are expressed as the percentage increase in cell number 6 days after treatment. (□) Control; (▤) $10^{-6}$ M E2; (■) MTX; (▨) MTX + $10^{-6}$ M E2.

Table III shows that substituting the poorly oestrogenic α-isomer (αE2), for E2 failed to result in potentiation of the anti-proliferative effects of MTX towards MCF-7 cells. Despite a slight, although non-significant, reduction in proliferative capacity during continuous exposure to $10^{-6}$ M αE2 alone, the effects of MTX were unaltered in the presence of the α-isomer. $10^{-6}$ M αE2 alone significantly inhibited proliferation of MDA-MB-436 cells (Table III), whilst a combination of αE2 and MTX resulted in partial reversal of the effect of the antimetabolite, although, in contrast to the results obtained with E2, this did not reach significance.

**Table III** The effect of αE2 ($10^{-6}$M) on the antiproliferative action of MTX towards MDA-MB-436 and MCF-7 cells.

| | % Change in cell no. (day 0 to day 6) | |
|---|---|---|
| | *MCF-7* | *MDA-MB-436* |
| Control | 712±104 | 425±35 |
| $10^{-6}$M αE2 | 550±70[a] | 327±28[b] |
| MTX (1) | 584±118 | 258±22 |
| MTX (1)+αE2 | 546±114[c] | 302±52[c] |
| MTX (2) | 368±48 | 113±23 |
| MTX (2)+αE2 | 394±52[c] | 132±10[c] |

MTX (1) and MTX (2) are the lower and higher doses of the drug respectively as described in **Figure 3**.
[a]$P>0.05$ with respect to control
[b]$P<0.01$ with respect to control
[c]$P>0.1$ with respect to MTX treatment alone (Student's "*t*" test).

### Discussion

The results presented demonstrate that E2 exposure can markedly influence the cytotoxicity of MTX towards human breast cancer cells growing *in vitro*. Furthermore E2 protects a non-E2 responsive breast cancer cell line from the effects of MTX whilst potentiating the drug's action towards an E2 responsive line. These modulating influences become significant at pharmacological concentrations of the hormone ($10^{-6}$M). It is probable that the ability of $10^{-6}$M E2 to partially reverse the anti-metabolic and anti-proliferative effects of MTX in the MDA-MB-436 cell line (Figures 2 and 3) is largely a consequence of the hormone's ability to reduce intra-cellular steady state MTX levels. These levels were reduced by 18 and 22% 24 and 48 h after drug treatment respectively (Table II). We are unable to fully explain our findings that E2 potentiates the effects of MTX in the MCF-7 cell line. Table II demonstrates that $10^{-6}$M E2 co-treatment reduces intracellular MTX levels in the E2 responsive line to a similar extent to the observed in the MDA-MB 436 line. Nevertheless the net result of combined drug-hormone treatment was increased MTX toxicity (Figures 2 and 3). Weichselbaum *et al.* (1978), reported that physiological concentrations of E2 enhanced the anti-proliferative action of cytosine arabinoside in MCF-7 cells. However, high concentrations ($10^{-7}$M) failed to produce this effect, an observation consistent with the author's findings that pharmacological concentrations of the hormone slightly decreased cell proliferative rate. Although we consistently observe marked stimulation of the incorporation of DNA precursors by a wide range of E2 concentrations (Figure 1), the effect of E2 on cell proliferative rate was somewhat variable. Although increased cell growth has been observed under our experimental conditions, Figure 3 shows that E2 potentiation of MTX action can occur in the absence of any demonstrable mitogenic effect of the hormone. Considerable variation in the biological response of MCF-7 cells to E2 has been reported and may reflect the influence of culture conditions (Page *et al.*, 1983), growth rate (Jakesz *et al.*, 1984), or the existence of different sublines (Katzenellenbogen *et al.*, 1984). The ability of E2 to increase the anti-metabolic and anti-proliferative action of MTX towards the MCF-7 line reported here is therefore unlikely to be solely the result of an E2 induced increase in cell proliferative capacity. It is possible that the increase in the rate of incorporation of [$^3$H]UdR into DNA of MCF-7 cells resulting from E2 treatment (Figure 1) may reflect an increase in the proportion of cells in S phase (Weichselbaum *et al.*, 1978), which might be expected to potentiate the cytotoxicity of a phase specific agent such as MTX. However it is equally possible that the potentiation observed is a consequence of E2 induced perturbations of other biochemical processes not here identified. Whatever the potentiating mechanism involved it is clear that it is sufficient to overcome the reduction of intracellular MTX levels concurrently caused by the hormone.

The proposal that the modulating effects of E2 on MTX toxicity in MCF-7 cells are mediated via the oestrogen receptor is supported by our observation that αE2 fails to mimic the action of the more biologically active β-isomer (Table III). Unlike E2, the α-isomer fails to stimulate DNA synthesis (Lippman *et al.*, 1976) and we have found that it is unable to reverse the effects of tamoxifen in MCF-7 cells (unpublished observations).

We have previously suggested that the ability of E2 to reduce intracellular MTX levels is unlikely to be a receptor mediated event (Clarke *et al.*, 1983). In a separate study (manuscript in preparation) we have observed that $10^{-6}$M E2 treatment results in a decrease in membrane fluidity in both cell lines, as determined by changes in the steady state fluorescence of a membrane associated probe, diphenylhexatriene. It is possible that such changes in lipid packing might result in a decrease in mobility of the MTX cell membrane transport protein complex.

If the interactions between E2 and MTX reported here occur *in vivo* then a combination of drug and hormone in a predominantly receptor negative tumour might be expected to decrease the efficacy of MTX. Whilst the combination may be of value in a receptor positive tumour, human breast cancer almost certainly consists of cell populations heterogeneous with respect to E2 receptor content, and receptor content may change during the course of treatment. The net result of combination treatment of such tumours may therefore be difficult to predict.

The authors wish to thank Margaret Andrews, Department of Surgery, Royal Victoria Hospital, Belfast, for carrying out the hormone receptor analyses. The generous financial support of Action Cancer, Northern Ireland, is gratefully acknowledged.

**References**

BRUNNER, K.W., SONNTAG, R.W., ALBERTO, P. & 4 others. (1977). Combined chemo- and hormonal therapy in advanced breast cancer. *Cancer*, **39,** 2923.

CARTER, S.K. (1981). The interpretation of trials: combined hormonal therapy and chemotherapy in disseminated breast cancer. *Breast Cancer Res. Treat.*, **1,** 43.

CLARKE, R., VAN DEN BERG, H.W. & MURPHY, R.F. (1983). Reduction of the antimetabolic and antiproliferative effects of methotrexate by 17-$\beta$-oestradiol in a human breast carcinoma cell line, MDA-MB-436. *Eur. J. Cancer Clin. Oncol.*, **19,** 19.

JAKESZ, R., SMITH, C.A., AITKIN, S. & 4 others. (1984). Influence of cell proliferation and cell cycle phase on expression of estrogen receptor in MCF-7 breast cancer cells. *Cancer Res.*, **44,** 619.

KATZENELLENBOGEN, B.S., NORMAN, M.J., ECKERT, R.L., PELTZ, S.W. & MANGEL, W.F. (1984) Bioactivities, estrogen receptor interactions and plasminogen activator-inducing activities of Tamoxifen and Hydroxy-tamoxifen isomers in MCF-7 human breast cancer cells. *Cancer Res.*, **44,** 112.

LIPPMAN, M., BOLAN, G. & HULL, K. (1976). The effects of oestrogens and anti-oestrogens on hormone responsive human breast cancer in long-term tissue culture. *Cancer Res.*, **36,** 4595.

PAGE, M.J., FIELD, J.K., EVERETT, N.P. & GREEN, C.D. (1983). Serum regulation of the estrogen responsiveness of the human breast cancer cell line MCF-7. *Cancer Res.*, **43,** 1244.

PILCHON, M.F. & MILGROM, E. (1977). Characterisation and assay of progesterone receptors in human mammary carcinoma. *Cancer Res.*, **37,** 464.

SHAFIE, S. & BROOKES, S.C. (1979). Characteristics of the dextran-coated charcoal assay for oestradiol receptor in breast cancer preparations. *J. Lab. Clin. Med.*, **94,** 784.

VAN DEN BERG, H.W. & BALL, C.R. (1972). The effect of methylazoxymethanol acetate on DNA synthesis and cell proliferation of synchronous HeLa cells. *Mutat. Res.*, **16,** 381.

WEICHSELBAUM, R.R., HELLMAN, S., PRIOR, A.J., NOVE, J.J. & LITTLE, J.B. (1978). Proliferation kinetics of a human breast cancer line in vitro following treatment with 17 $\beta$-estradiol and 1-B-D-Arabinofuranosylcytosine. *Cancer Res.*, **38,** 2339.


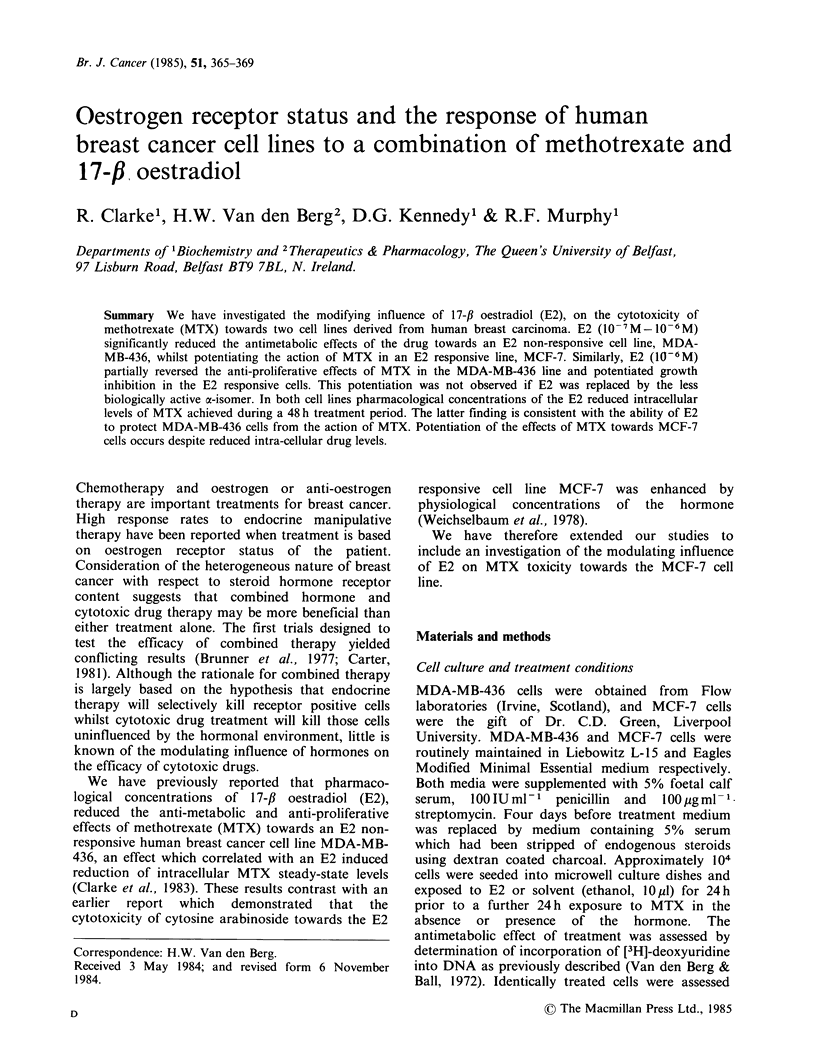

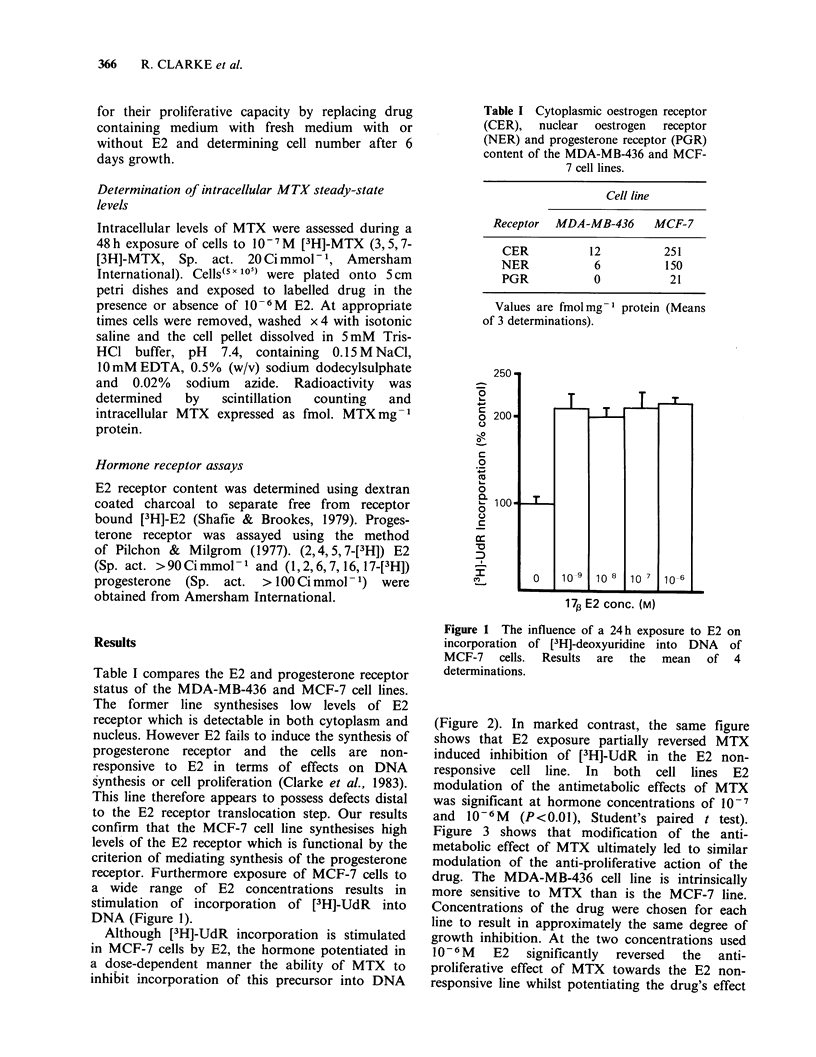

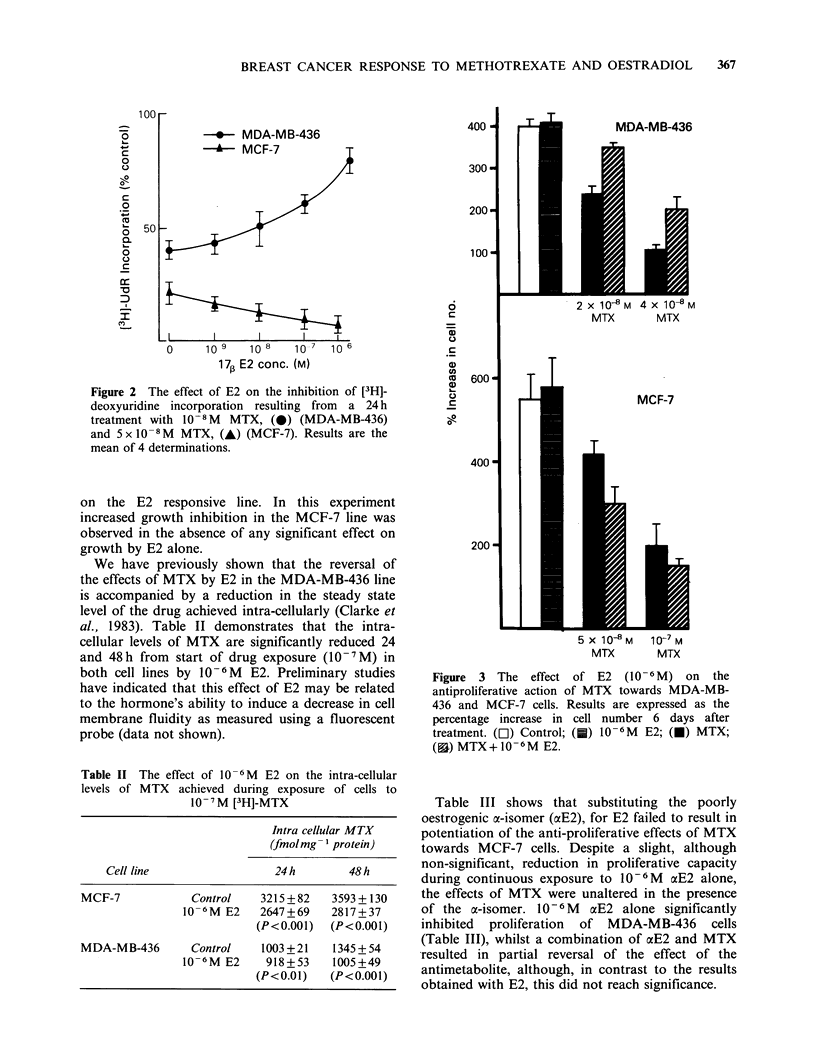

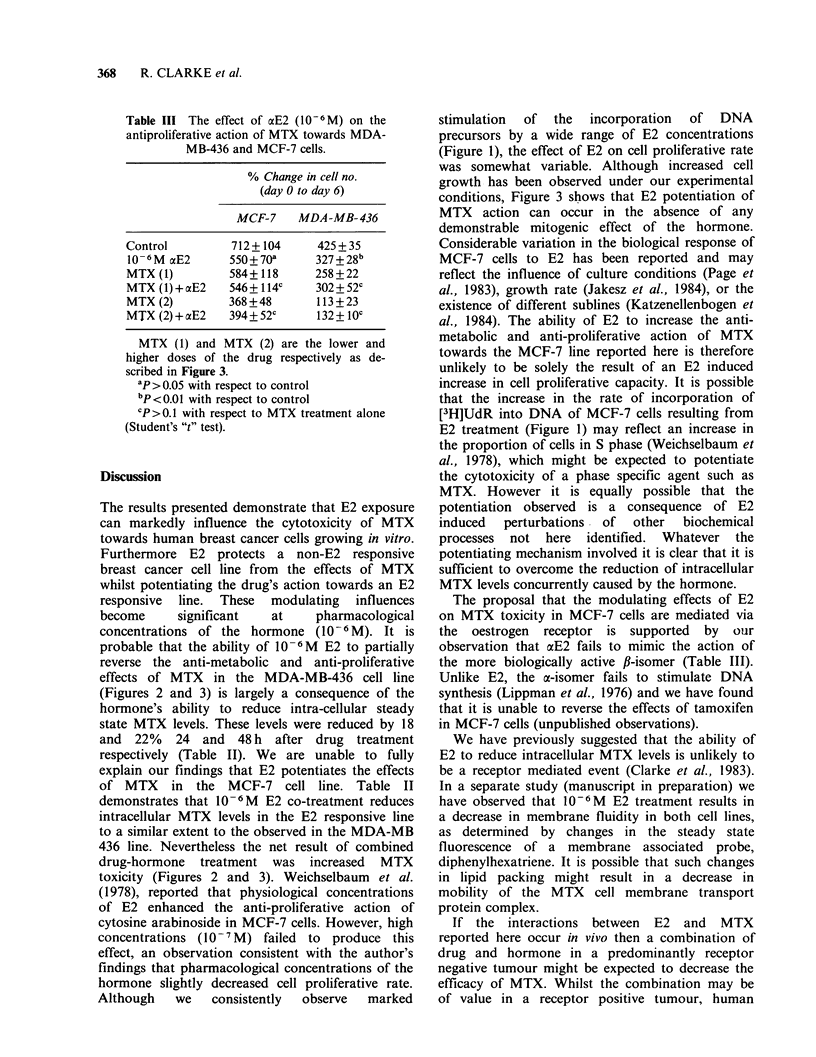

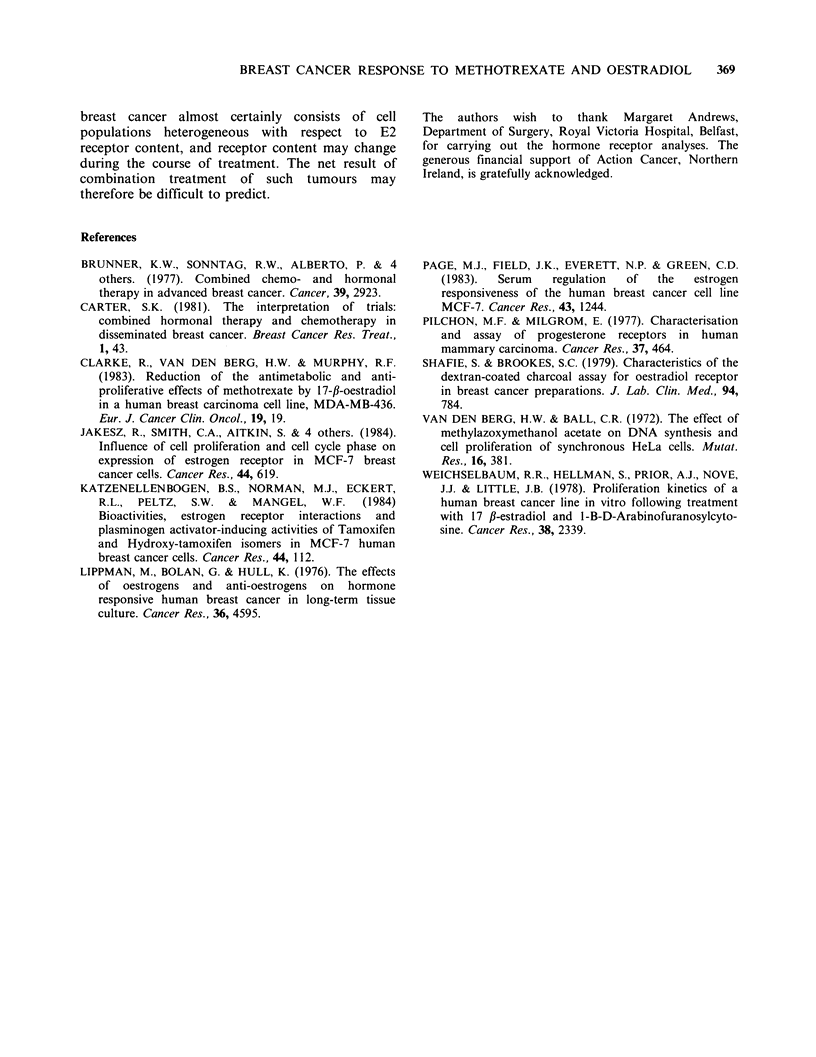


## References

[OCR_00530] Brunner K. W., Sonntag R. W., Alberto P., Senn H. J., Martz G., Obrecht P., Maurice P. (1977). Combined chemo- and hormonal therapy in advanced breast cancer.. Cancer.

[OCR_00535] Carter S. K. (1981). The interpretation of trials: combined hormonal therapy and chemotherapy in disseminated breast cancer.. Breast Cancer Res Treat.

[OCR_00541] Clarke R., Van den Berg H. W., Kennedy D. G., Murphy R. F. (1983). Reduction of the anti-metabolic and anti-proliferative effects of methotrexate by 17 beta-oestradiol in a human breast carcinoma cell line, MDA-MB-436.. Eur J Cancer Clin Oncol.

[OCR_00548] Jakesz R., Smith C. A., Aitken S., Huff K., Schuette W., Shackney S., Lippman M. (1984). Influence of cell proliferation and cell cycle phase on expression of estrogen receptor in MCF-7 breast cancer cells.. Cancer Res.

[OCR_00554] Katzenellenbogen B. S., Norman M. J., Eckert R. L., Peltz S. W., Mangel W. F. (1984). Bioactivities, estrogen receptor interactions, and plasminogen activator-inducing activities of tamoxifen and hydroxy-tamoxifen isomers in MCF-7 human breast cancer cells.. Cancer Res.

[OCR_00562] Lippman M., Bolan G., Huff K. (1976). The effects of estrogens and antiestrogens on hormone-responsive human breast cancer in long-term tissue culture.. Cancer Res.

[OCR_00568] Page M. J., Field J. K., Everett N. P., Green C. D. (1983). Serum regulation of the estrogen responsiveness of the human breast cancer cell line MCF-7.. Cancer Res.

[OCR_00574] Pichon M. F., Milgrom E. (1977). Characterization and assay of progesterone receptor in human mammary carcinoma.. Cancer Res.

[OCR_00579] Shafie S., Brooks S. C. (1979). Characteristics of the dextran-coated charcoal assay for estradiol receptor in breast cancer preparations.. J Lab Clin Med.

[OCR_00585] Van den Berg H. W., Ball C. R. (1972). The effect of methylazoxymethanol acetate on DNA synthesis and cell proliferation of synchronous HeLa cells.. Mutat Res.

[OCR_00591] Weichselbaum R. R., Hellman S., Piro A. J., Nove J. J., Little J. B. (1978). Proliferation kinetics of a human breast cancer line in vitro following treatment with 17beta-estradiol and 1-beta-D-arabinofuranosylcytosine.. Cancer Res.

